# Supplementary material for: Predicting visual function by interpreting a neuronal wiring diagram
Source: Nature. 2024 Oct 2;634(8032):113–23. doi: 10.1038/s41586-024-07953-5 (PMC11446822; doi:10.1038/s41586-024-07953-5)
Supplement: Supplementary file 6 — CRF and ERF predictions for individual TmY4 and TmY9 cells. Analogous to Supplementary Data 3, but for TmY target types. Shown are the top four monosynaptic pathways, the strongest pathway passing through each of the top ten intermediary types (ranking from Extended Data Fig. 7), and the trisynaptic pathway Tm1–TmY–Dm3–TmY (see the section entitled Prediction of spatial normalization). [file 41586_2024_7953_MOESM6_ESM.zip › DataS4/TmY9q/720575940632347022.pdf]

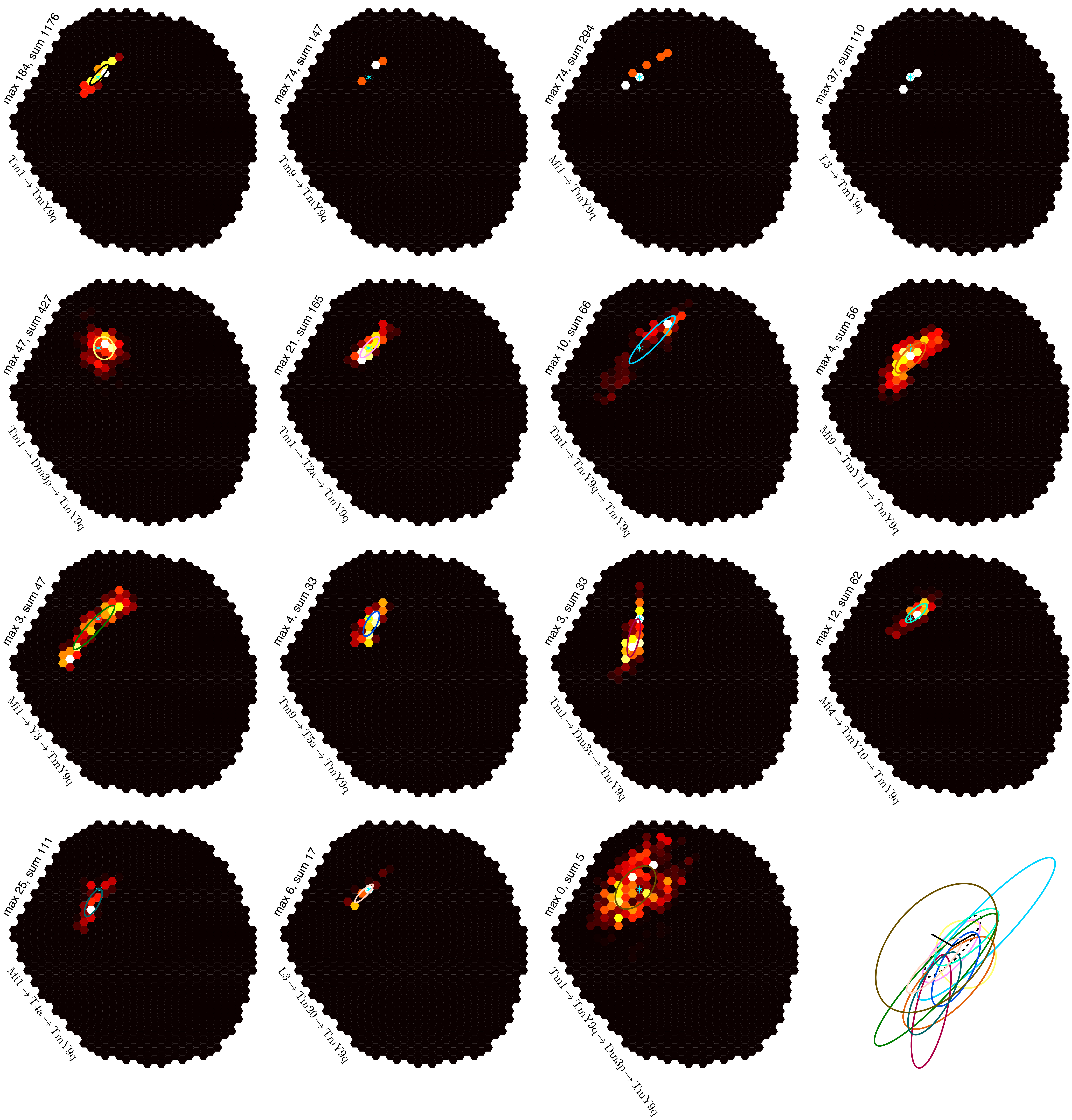

max 184, sum 1176
Tm1 → TmY9q
max 74, sum 147
Tm9 → TmY9q
max 74, sum 294
Mi1 → TmY9q
max 37, sum 110
L3 → TmY9q
max 47, sum 427
Tm1 → Dm3p → TmY9q
max 21, sum 165
Tm1 → T2a → TmY9q
max 10, sum 66
Tm1 → TmY9q → TmY9q
max 4, sum 56
Mi9 → TmY11 → TmY9q
max 3, sum 47
Mi1 → Y3 → TmY9q
max 4, sum 33
Tm9 → T5a → TmY9q
max 3, sum 33
Tm1 → Dm3v → TmY9q
max 12, sum 62
Mi4 → TmY10 → TmY9q
max 25, sum 111
Mi1 → T4a → TmY9q
max 6, sum 17
L3 → Tm20 → TmY9q
max 0, sum 5
Tm1 → TmY9q → Dm3p → TmY9q
